# Fine mapping of the hereditary haemorrhagic telangiectasia *(HHT)3 *locus on chromosome 5 excludes VE-Cadherin-2, Sprouty4 and other interval genes

**DOI:** 10.1186/2040-2384-2-15

**Published:** 2010-08-11

**Authors:** Fatima S Govani, Claire L Shovlin

**Affiliations:** 1 NHLI Cardiovascular Sciences, Imperial College London, UK, Hammersmith Campus, Du Cane Rd, London W12 0NN, UK

## Abstract

**Background:**

There is significant interest in new loci for the inherited condition hereditary haemorrhagic telangiectasia (HHT) because the known disease genes encode proteins involved in vascular transforming growth factor (TGF)-β signalling pathways, and the disease phenotype appears to be unmasked or provoked by angiogenesis in man and animal models. In a previous study, we mapped a new locus for HHT (*HHT3*) to a 5.7 Mb region of chromosome 5. Some of the polymorphic markers used had been uninformative in key recombinant individuals, leaving two potentially excludable regions, one of which contained loci for attractive candidate genes encoding VE Cadherin-2, Sprouty4 and FGF1, proteins involved in angiogenesis.

**Methods:**

Extended analyses in the interval-defining pedigree were performed using informative genomic sequence variants identified during candidate gene sequencing. These variants were amplified by polymerase chain reaction; sequenced on an ABI 3730xl, and analysed using FinchTV V1.4.0 software.

**Results:**

Informative genomic sequence variants were used to construct haplotypes permitting more precise citing of recombination breakpoints. These reduced the uninformative centromeric region from 141.2-144 Mb to between 141.9-142.6 Mb, and the uninformative telomeric region from 145.2-146.9 Mb to between 146.1-146.4 Mb.

**Conclusions:**

The *HHT3 *interval on chromosome 5 was reduced to 4.5 Mb excluding 30% of the coding genes in the original *HHT3 *interval. Strong candidates VE-cadherin-2 and Sprouty4 cannot be *HHT3*.

## Background

Transforming growth factor (TGF)-β superfamily signalling is of fundamental importance to developmental and physiological regulation. In these pathways (reviewed in [1,2]), ligands such as TGF-βs, bone morphogenetic proteins (BMP)s, activins, nodals, growth/differentiation factors (GDF)s and inhibins bind to receptor complexes of paired type I and type II transmembrane receptor serine/threonine kinases. Activated type I receptors (ALKs 1-7) phosphorylate receptor-associated Smad proteins in complex-specific patterns [3-5]. There is increasing recognition of the role of alternative signalling pathways for particular ligands within designated cell types. In endothelial cells (ECs), signalling through the TGF-β type II receptor, TβRII, can be propagated not only through ALK-5 via SMAD2/3 as in other cell types, but also through ALK-1 via SMAD1/5/8, providing two mutually antagonistic pathways [6,7]. The transmembrane glycoprotein endoglin is an accessory TGF-β receptor, highly expressed on ECs, and is one factor modulating the balance between ALK-1 and ALK-5 pathways [8].

The inherited vascular condition hereditary haemorrhagic telangiectasia (HHT) [9] is of significant relevance to TGF-β signalling because the genes for endoglin, ALK-1 and SMAD4 (a co-Smad and downstream effector of the TGF-β signalling pathway), are mutated in different HHT families [10-12]. HHT is transmitted as an autosomal dominant trait due to a single mutation in either *ENG *encoding endoglin (HHT type 1); *ACVRL1 *encoding ALK-1 (HHT type 2) or *SMAD4 *(HHT in association with juvenile polyposis). Perturbation of TGF-β signalling pathways is therefore implicated in HHT pathogenesis.

HHT serves not only as a vascular model of aberrant TGF-β superfamily signalling, but also as a model of aberrant angiogenesis [13,14]. The abnormal blood vessels develop only in selected vascular beds (telangiectasia particularly in mucocutaneous and gastrointestinal sites; arteriovenous malformations (AVMs) most commonly in pulmonary, hepatic and cerebral circulations) [9,15]. At each site, only a small proportion of vessels are abnormal. The context in which HHT mutations are deleterious, when allowing apparently normal endothelial function for most vessels, now appear to be angiogenic in origin. Early studies modelling HHT in transgenic animals provided evidence of aberrant angiogenesis. Heterozygous mice developed HHT-like features; *Eng *and *Alk1 *null mice died by E11.5 with normal vasculogenesis but abnormal angiogenesis [7,16-20]. The zebrafish *violet beauregarde *(*vbg*), an *Alk1 *mutant, was also homozygous embryonic lethal, with mutant embryos displaying dilated cranial vessels attributed to an increased number of endothelial cells [21]. More recent studies have demonstrated that an *Alk1 *deletion in adult mouse subdermal blood vessels resulted in AVM formation in wounded areas displaying angiogenesis [22]; that angiogenic stimuli promoted AVM formation in endothelial specific endoglin knockout mice, accompanied by an abnormal increase in EC proliferation [23] which was also observed in Eng^-/- ^mouse embryonic ECs [8], and that *Alk1 *knockout mice had defective smooth muscle differentiation and recruitment and excessive angiogenesis [7]. These data from animal models have been accompanied by clinical reports that Bevacizumab, an antibody against vascular endothelial growth factor (VEGF)-A, and thalidomide, appear to have efficacy in treating clinical manifestations of HHT in man [24,25].

A current model to explain these observations, discussed in more detail in [26], is based on the EC-mural cell axis defined by Sato and Rifkin [27]. In angiogenesis, HHT mutations (endoglin and ALK-1) appear to impair recruitment of mural cells to the angiogenic sprout [7,28] at least in part via reduced EC secretion of TGF-β1 [29,30] and/or reduced TGF-β1 induced responses [7,29] resulting in defective mural cell stabilisation of the nascent vessel and persistent, excessive, EC proliferation. Thalidomide, which induced vessel maturation in Eng^+/- ^mice which normally suffer from excessive angiogenesis, appears to target mural cell recruitment, by increasing endothelial expression of PDGF-B at the endothelial tip cell, thus facilitating recruitment of pericytes that express PDGFR-b, associated with increasing pericyte proliferation [25].

**Figure 1:**
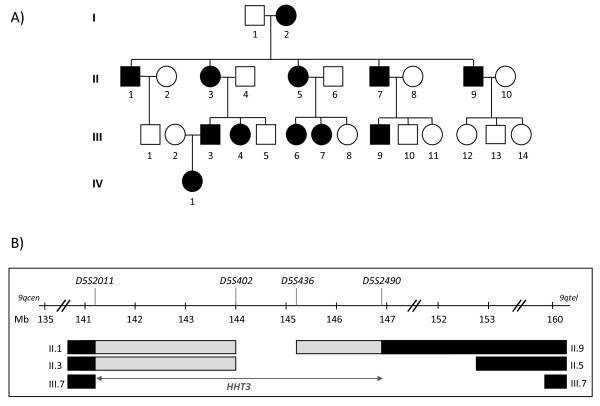
**Previous *HHT3 *interval**. **A) ***HHT3 *linked Family S pedigree (black symbols represent HHT-affected individuals; white symbols represent unaffected individuals; squares represent males and circles represent females. Individuals are denoted by their generation number, such that the oldest male is I.1, oldest female I.2). **B) **Published *HHT3 *interval on chromosome 5 between microsatellite markers *D5S2011 *and *D5S2490 *[35], identifying the region shared by all affected individuals (open interval between *D5S402 *and *D5S436*); regions where the designated individuals had inherited their affected parent's non disease-gene bearing allele (black bars), and regions in which the transition from the non disease-gene bearing allele (black bars) to disease-gene bearing allele (open interval) had occurred, but the exact sites of the meiotic recombination event was not definable using the markers studied in [35] (grey bars; uninformative markers). It was possible that the interval could be reduced to a minimum of *D5S402-D5S436*, according to where the recombination breakpoints had occurred in individuals II.1, II.3 and II.9. cen: centromere; tel: telomere; Mb: mega bases.

Further HHT genes were therefore predicted to identify new components or regulators of TGF-β/BMP signalling pathways of particular relevance to angiogenesis. More than 80% of HHT patients carry a sequence variation in *endoglin *or *ALK-1 *[31], most (but not all [32,33]) disease-causing, whilst 1-2% carry a mutation in *SMAD4 *[34]. HHT patients in whom no mutations have been detected in *endoglin*, *ALK-1 *or *SMAD4 *may have mutations in unsequenced intronic regions, or undetected large rearrangements of these genes, but importantly, they may have a mutation in a different gene. A third gene for pure HHT, *HHT3*, was mapped in our laboratory, using the pedigree illustrated in Figure 1A, to chromosome 5q [35] (Figure 1B), and a fourth, *HHT4*, to chromosome 7p [36].

The published chromosome 5 *HHT3 *interval which contains the *HHT3 *gene, is 5.7 Mb long, between microsatellite markers *D5S2011 *and *D5S2490 *(Figure 1B) [35]. This interval contains 38 genes according to the Ensembl genome browser http://www.ensembl.org. There were two groups of potentially very attractive candidate genes. The first encode proteins involved in angiogenesis: *PCDH12 *and *SPRY4 *(discussed further below), and *FGF1 *encoding the proangiogenic fibroblast growth factor 1 [37]). A second group of interval genes encode proteins involved in signalling by serine/threonine kinases: *PPP2R2B *which encodes the B regulatory subunit of protein phosphatase 2A [38], and *STK32A*, encoding a serine/threonine kinase [39]. While these genes had been sequenced with no disease-causing mutations found ([35], Govani and Shovlin unpublished), this did not exclude the genes as disease-causing, due to the possibility of undetected intronic mutations. Importantly *PCDH12 (*141.3 Mb), *SPRY4 *(141.6 Mb) and *FGF1 *(142 Mb) were at the centromeric extreme of the *HHT3 *interval, and *PPP2R2B *(146.4 Mb) and *STK32A *(146.6 Mb) at the telomeric extreme, all within potentially excludable regions of the interval [35].

Excluding any of these genes from the *HHT3 *interval was important not only to assist the identification of *HHT3 *itself, but also to inform scientists working on the relevant proteins, particularly VE-Cadherin-2 (encoded by *PCDH12*) and Sprouty4 (encoded by *SPRY4*): VE-Cadherin-2, primarily expressed on ECs [40], was shown to be down-regulated in HHT1 and HHT2 blood outgrowth ECs [41] with a role in angiogenesis [42]. Furthermore, a close family member (VE-cadherin), associates with endoglin and ALK-1 in cell surface complexes, and promotes TGF-β1 signalling by facilitating the recruitment of TGF-β receptor II (TβRII) into the active signalling complex [43]. Sprouty family members are involved in branching morphogenesis [44]. Overexpression of *Spry4 *in ECs of embryonic day (E)9 mice inhibited angiogenesis [45], whereas surviving *Spry4 *knockout mice [46] demonstrated enhanced angiogenesis [47].

In this study we report the fine mapping of the published *HHT3 *interval using genomic sequence variants detected during candidate gene sequencing, and exclusion of key genes.

## Methods

### Pedigree

Ethics approval was obtained from the Multicentre Research Ethics Committee for Scotland (MREC/98/0/42; 07/MRE00/19), by the Hammersmith, Queen Charlotte's, Chelsea, and Acton Hospital Research Ethics Committee (LREC 99/5637M) and in 2007 by the Hammersmith Hospitals Trust (SHOV1022, 2007). The study is registered on http://www.clinicaltrials.gov (NCT00230620). The pedigree (Family S, Figure 1A) and DNA extractions were as described in [35].

### PCR and sequencing

Oligonucleotide primers were ordered from Eurofins MWG-Biotech and amplified in informative members of Family S by polymerase chain reaction (PCR). Genomic primers spanning exons and > 30 bp of flanking intronic sequences were amplified by PCR using *Phusion Hotstart *(New England Biolabs) or *AmpliTaq Gold *(Applied Biosystems) DNA polymerases. Reactions were performed as recommended by the manufacturers using 35-40 cycles of denaturation, annealing (at primer specific Ta) and extension. PCR products were treated with an ExoSAP mix containing SAP enzyme and buffer (Promega) and ExoI enzyme (New England Biolabs) as previously described [48]; gel purified with Wizard SV gel and PCR clean-up system (Promega) following the manufacturer's protocol; or gel purified and ethanol precipitated using Costar Spin-X column centrifuge tubes [49] followed by ethanol precipitation [50]. Purified PCR products were sequenced in 10 μl reactions with 25 ng (≅ 3.2 pM) of the sequencing primer on an ABI 3730xl DNA analyzer (MRC CSC DNA core laboratory, Imperial College London, Hammersmith). The results were analysed using FinchTV V1.4.0 software (Geospiza, Inc).

### Fine mapping

**Figure 2:**
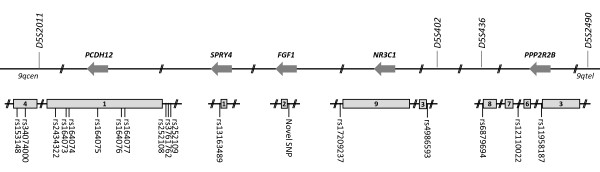
**Informative genomic sequence variants used in fine mapping**. Locations of genomic variants where individual I.2 was heterozygous, potentially allowing her disease associated and non-disease associated alleles to be tracked. Sequence variants are illustrated in relation to candidate gene exons (grey boxes) and original microsatellite markers. By chance, all five genes are on the reverse strand of chromosome 5, designated by reverse arrows. *rs; *as on NCBI dbSNP.

**Figure 3:**
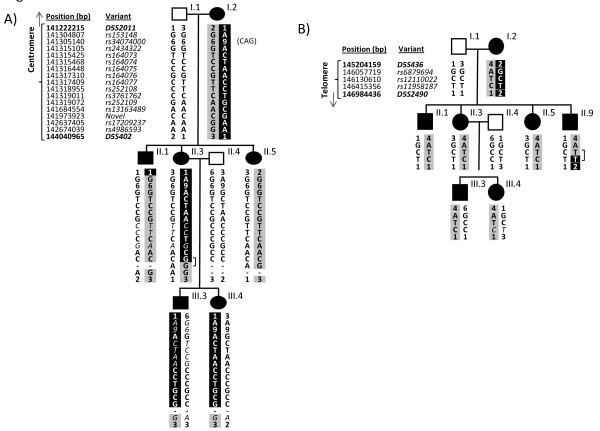
**Genomic variant haplotypes in interval-defining members of the pedigree**. **A) **Allele inheritance and haplotypes for the inheritance of the key microsatellite markers, and new genomic sequence variants, across *PCDH12, SPRY4, FGF1 *and *NR3C1 *genes in eight members of Family S. For each sequence variant, offspring inherit one allele from their mother and one from their father. Occasional alleles where it was not possible to determine parental origin (and which were therefore not used for haplotype construction) are denoted in non-bold italics. Individual II.5 demonstrates the disease haplotype across the interval, a haplotype which is now shared by II.1, but not II.3 and her descendants until *rs17209237 *in *NR3C1*. Therefore II.3 excludes the centromeric region between *rs153148 *and the novel SNP in *FGF1*. **B) **Haplotypes for the inheritance of the new genomic sequence variants across the *PPP2R2B *gene in nine members of Family S. Affected individuals II.1, II.3, II.5, III.3 and III.4 demonstrate the disease haplotype which is not shared by II.9 at *rs 11958187*, but is shared at *rs12110022, rs 6879694*, and the remainder of the *HHT3 *interval. Therefore II.9 excludes *rs11958187 *and beyond from the telomeric extreme of the interval.]: uninformative regions; bp: base pairs.

Non-disease causing genomic sequence variants found during *HHT3 *candidate gene sequencing were used to fine map the interval, by providing further polymorphisms to track known recombination events through the uninformative part of the interval. Previously studied microsatellite polymorphisms had not been able to distinguish between disease and non-disease alleles (Figure 1B; grey bars), predominantly due to homozygosity in individual I.2 (Figure 1A). New sequence variants where individual I.2 was heterozygous (Figure 2) were sequenced in the interval-defining members of the pedigree (Figure 3). Adjacent genes (such as *NR3C1*, mutations in which cause glucocorticoid resistance [51]), were also sequenced to identify further polymorphisms to define the precise centromeric recombination event. The alleles inherited by each pedigree member were then assessed and assigned to maternal or paternal origin using Mendelian principles. Haplotypes were then constructed to minimise the number of recombination events that would be required to generate the segments of DNA in each offspring. NCBI dbSNP http://www.ncbi.nlm.nih.gov/projects/SNP/ and SNPbrowser™ V3.5 (Applied Biosystems) were used to determine if the genomic sequence variants were previously known (when they were allocated the relevant *rs *dbSNP RefSNP label), or novel.

For confirmations of genotypes, SNPs *rs153148, rs34074000, rs164075, rs164077, rs6879694, rs12110022 *and *rs11958187 *were sequenced in two separate PCR reactions in two or more key individuals.

### Genome analysis

Chromosome 5 positions of genomic sequence variants are based on NCBI Reference build 36 (hg18) assembly.

## Results

The *HHT3 *interval [35] was defined by recombination events in three affected individuals of Family S, II.1, II.3, and II.9 (Figure 1). At each end of the interval, there were regions illustrated by grey bars, in which it was not possible to state which maternal chromosome 5 allele sequences these individuals had inherited, due to homozygosity in individual I.2. New non-microsatellite genomic sequence variants were identified during candidate gene sequencing and analysed to assess if they could be used to track the inheritance of the sequences derived from the two different alleles of chromosome 5 from individual I.2 in the regions between *D5S2011 *and *D5S402*, and between *D5S436 *and *D5S2490*. A total of 89 genetic sequence variations were found (75 SNPs, 4 indels and 10 repeats) of which 73 were present on dbSNP (2010, build 131). Eighteen potentially informative genomic sequence variants where I.2 was a heterozygote (seventeen SNPs; one triplet repeat) were identified within the regions of interest (Figure 2).

Figure 3 tracks the inheritance of alleles at these SNPs and triplet repeat in the centromeric (Figure 3A), and telomeric (Figure 3B) extremes of the interval. Although I.2 shared three genotypes with I.1, it was possible to definitely state which of I.2's alleles had been inherited by each of their children at all sites except *rs164077*, *rs252108 *and *rs252109 *where genotypes were shared. Sites where the inheritance of the affected parent's alleles could definitely be determined were used to generate haplotypes.

As shown in Figure 3A, sequencing 15 of these markers allowed better definition of the centromeric regions where II.1 and II.3 had inherited the disease-gene associated allele, and indicated that the site of the recombination breakpoint differed in the two individuals. In individual II.1, the breakpoint had occurred close to *D5S2011 *such that he had inherited the maternal disease allele from *rs153148 *(within *PCDH12*) at 141.3 Mb and onwards. However, in individual II.3, the recombination event had occurred further into the *HHT3 *interval: II.3 continued to inherit a different maternal allele to her affected siblings from *rs153148 *to a novel SNP (within *FGF1*) at 141.9 Mb. By 142.6 Mb (*rs17209237 *and *rs4986593 *within *NR3C1*), II:3 had inherited the same maternal allele as her affected siblings, siting the recombination event between 141.9 Mb and 142.6 Mb (novel SNP and *rs17209237*)

**Figure 4:**
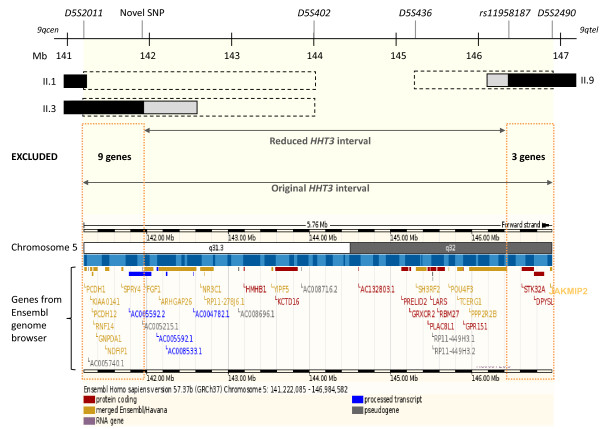
**The refined *HHT3 *interval**. The original *HHT3 *interval was between microsatellite markers *D5S2011 *and *D5S2490*, and uninformative between *D5S2011-D5S402 *and *D5S2490-D5S436 *(black dotted bars) in key individuals II.1, II.3 and II.9 [35]. Informative genomic variations used in fine mapping reduced the extent of the uninformative regions (grey bars, see Figure 1B). The refined *HHT3 *interval is sited between a novel SNP within *FGF1 *intron 1, and *rs11958187 *within *PPP2R2B *intron 4. Black bars indicate the definite recombination events that defined the interval in the denoted individuals. Fine mapping, by reducing the interval, excluded 12 interval genes as shown in the lower Ensembl genome browser image of candidate genes (genes no longer in the interval are denoted by orange dotted boxes; Mb: mega bases).

These data in individual II.3 indicated that in addition to the definite *D5S402*-*D5S436 *region (Figure 1B), the maximal centromeric extent of the disease gene-bearing chromosome 5 that could have been inherited by all affected individuals in the pedigree extended not from *D5S2011 *at 141.2 Mb, but from the *FGF *SNP at 141.9 Mb (Figure 4). This reduction of the uninformative centromeric region by 0.7 Mb excluded 9 Ensembl interval genes (Figure 4). *PCDH12 *and *SPRY4 *were amongst the group of genes excluded (Figure 4).

The telomeric border of the *HHT3 *interval was defined by a recombination event in affected individual II.9. This individual had inherited a non-disease allele at microsatellite markers up to and including *D5S2490 *(146.9 Mb), and was uninformative at microsatellite markers up to *D5S436 *(145.2 Mb) [35] (Figure 1B). Figure 3B demonstrates the results of sequencing genomic sequence variants within genes in the potentially excludable telomeric extreme of the interval. II.9 had inherited the maternal disease allele from *rs6879694 *to *rs12110022*, but a different maternal allele to his affected siblings at *rs11958187 *(within *PPP2R2B*). This sited the recombination event between 146.1-146.4 Mb, excluding a further 3 Ensembl interval genes; *JAKMIP2, DPYSL*, and *STK32A *(Figure 4).

## Discussion

Identification of further HHT genes is predicted to provide new insights into TGF-β/BMP signalling and angiogenesis pathways. The previously published *HHT3 *interval included several highly attractive candidate genes. As further evidence accumulated regarding the roles of particularly VE-cadherin and Sprouty family members, the possibility that one of these may be mutated in HHT increased their interest to endothelial cell biologists. While these genes had been sequenced with no disease-causing mutations found in the coding or untranslated regions in affected members of the chromosome 5 linked family ([35], Govani and Shovlin unpublished), such sequencing did not exclude the genes as disease-causing, due to the possibility of undetected mutations in intronic or poorly characterised regulatory regions.

It was recognised that fine mapping of the genomic variants found during candidate gene sequencing could help define the critical recombination events and exclude certain genes, including those for VE-cadherin-2 and Sprouty4. Hence candidate gene sequence variants were examined in the interval-defining members of the Family S pedigree that included the crucial individuals whose recombination events had defined the centromeric and telomeric borders of the *HHT3 *interval.

To optimise PCR fidelity, candidate genes and genomic sequence variants were amplified with *AmpliTaq Gold *or *Phusion Hotstart *DNA polymerases, enzymes that are inactive until they undergo heat activation to generate polymerase activity, thus providing an automated 'hot start' [52-54]. To further confirm key genotypes, SNPs *rs153148, rs34074000, rs164075, rs164077, rs6879694, rs12110022 *and *rs11958187 *were sequenced in two separate PCR reactions in two or more key individuals.

Confirmed genotypes allowed construction of haplotypes across the centromeric and telomeric extremes of the *HHT3 *interval. The recombination events were then sited more precisely, leading to findings which excluded 12 interval genes such as strong candidates *PCDH12*, *SPRY4 *and *STK32A*. *FGF1 *and *PPP2R2B *could not be fully excluded as the centromeric and telomeric defining recombination events (SNPs 'novel' and *rs11958187*) are within the introns of both genes. It should be noted that the siting of a recombination breakpoint in a single affected member of the pedigree does not carry any pathogenic implications for the genes in question, unlike disease-causing chromosomal rearrangements when the causative rearrangement is shared by all of the affected individuals in the pedigree.

The original interval contained 38 genes according to the Ensembl genome browser. Twelve have been excluded in this study. The 26 genes that remain include 10 pseudogenes or processed transcripts with no known protein products; two genes of potential functional relevance to angiogenesis or TGF-β signalling (*FGF1 *and *PPP2R2B*); three protein coding genes associated with other genetic disease (*ARHGAP26*; *NR3C1 *and *POU4F3*) and 11 protein coding genes not associated with a genetic disease but whose gene product had no obvious function in TGF-β signalling and/or angiogenesis (*HMHB1; YIPF5; KCTD16; PRELID2; LARS; TCERG1; SH3RF2; GRXCR2; RBM27; PLAC8L1 *and *GPR151*).

## Conclusions

Further examination of key recombination events in a known *HHT3 *pedigree has reduced the published *HHT3 *gene interval. Crucially, the *PCDH12 *and *SPRY4 *genes were amongst twelve genes no longer in the reduced interval. Therefore VE-cadherin-2 and Sprouty4, proteins of significant interest to angiogenesis researchers due to their respective roles in pathophysiological angiogenesis and relationship to known interactors with the HHT gene products, are not mutated in *HHT3 *[55]. Reduction of the interval has facilitated *HHT3 *gene identification studies.

## Abbreviations

HHT: hereditary haemorrhagic telangiectasia; TGF-β: transforming growth factor-beta; ALK: activin receptor-like kinase; AVMS: arteriovenous malformations; SNP: single nucleotide polymorphism; PCR: polymerase chain reaction; ECS: endothelial cells.

## Competing interests

The authors declare that they have no competing interests.

## Authors' contributions

CLS and FSG designed the study. All experimental work was performed by FSG, with advice from CLS. The manuscript was co-written by CLS and FSG; both authors approved the final version.
